# Oxidative Stress: Role and Response of Short Guanine Tracts at Genomic Locations

**DOI:** 10.3390/ijms20174258

**Published:** 2019-08-30

**Authors:** Anju Singh, Ritushree Kukreti, Luciano Saso, Shrikant Kukreti

**Affiliations:** 1 Nucleic Acids Research Lab, Department of Chemistry, University of Delhi (North Campus), Delhi 110007, India; 2 Department of Chemistry, Ramjas College, University of Delhi, Delhi 110007, India; 3 Academy of Scientific and Innovative Research (AcSIR), CSIR-Institute of Genomics and Integrative Biology (CSIR-IGIB) Campus, Delhi 110007, India; 4 Genomics and Molecular Medicine Unit, Institute of Genomics and Integrative Biology (IGIB), Council of Scientific and Industrial Research (CSIR), Mall Road, Delhi 110007, India; 5 Department of Physiology and Pharmacology “Vittorio Erspamer”, Sapienza University of Rome, P. le Aldo Moro 5, 00185 Rome, Italy

**Keywords:** oxidative stress, guanine base, G-quadruplex, 8-oxo-dG, reactive oxygen species, oxidation

## Abstract

Over the decades, oxidative stress has emerged as a major concern to biological researchers. It is involved in the pathogenesis of various lifestyle-related diseases such as hypertension, diabetes, atherosclerosis, and neurodegenerative diseases. The connection between oxidative stress and telomere shortening via oxidative guanine lesion is well documented. Telomeres are confined to guanine rich ends of chromosomes. Owing to its self-association properties, it adopts G-quadruplex structures and hampers the overexpression of telomerase in the cancer cells. Guanine, being the most oxidation prone nucleobase, when structured in G-quadruplex entity, is found to respond peculiarly towards oxidative stress. Interestingly, this non-Watson–Crick structural feature exists abundantly in promoters of various oncogenes, exons and other genomic locations. The involvement of G-quadruplex architecture in oncogene promoters is well recognized in gene regulation processes. Development of small molecules aimed to target G-quadruplex structures, have found to alter the overexpression of oncogenes. The interaction may lead to the obstruction of diseased cell having elevated level of reactive oxygen species (ROS). Thus, presence of short guanine tracts (Gn) forming G-quadruplexes suggests its critical role in oxidative genome damage. Present review is a modest attempt to gain insight on the association of oxidative stress and G-quadruplexes, in various biological processes.

## 1. Introduction

Indispensable role of Oxygen for living beings in biological system is undebatable. It is the element of utmost importance for the sustainability of life on earth. During respiration this oxygen gets reduced by the cells with concomitant generation of adenosine triphosphate (ATP) in the respiration process, occurring in the mitochondria. There are various reactive oxygen species identified in partially reduced form including superoxide anion (O_2_^•−^), hydrogen peroxide (H_2_O_2_), hydroxyl radical (OH^•^), and reactive nitrogen species (RNS) (nitric oxide etc.) produced continuously from organism as the byproducts of various biochemical and physiological processes. Simultaneously, antioxidants such as glutathione, arginine [1,2], creatine [3], zinc [4], and vitamin A are produced against these reactive species to combat and proper regulation of these ROS/RNS. The imbalance between ROS and its scavenging is known as oxidative stress. Interestingly, presence of these free radicals in optimum concentration is desired in various biological processes [5]. Overproduction of these ROS is found to be the main culprit for the imbalance and deterioration of cell components (nucleic acids, proteins, and lipids) via interacting potentially with said macromolecules. These ROS can cause oxidation of cellular macromolecules specifically to nucleic acids via generating single nucleobase-lesions, DNA strand breaks, cross-linking of inter- and intrastrand of nucleic acid bases, as well as protein-DNA cross-links. [6,7]. Recently a new term, ferroptosis, has also drawn much attention owing to its association with iron metabolism. In ferroptosis, soluble and lipid ROS are generated by involvement of enzymatic reactions catalyzed by transition metal iron. OS plays substantial role in ferroptosis as well as mitochondrial malfunction/dysfunction, leads to the neuronal cell death along with apoptosis [8]. Undeniably, oxidation is supposed to play a pivotal role in causing various skin diseases, aging, neurodegenerative diseases, and cancers [9,10,11,12,13,14,15].

Lesions resulting from the oxidation of nucleic acids lead to various types of mutations and obstruct DNA replication, transcription, translation, and subsequently altered gene expression and regulation [16,17,18]. DNA bases, specifically the guanine, are very much susceptible to oxidation due to having a low redox potential. Literature is rich in describing the promoters and telomeric regions of most genomes as G-rich [19,20]. It is now evident that owing to the self-association property of the guanine base, these guanine-rich sequences can adopt G-quartets resulting in a folded G-quadruplex structure. G-quadruplex, a non-Watson–Crick DNA structure, has drawn attention of scientific community due to its unique stability and polymorphic behavior. These structures are abundantly found in gene promoters of various oncogenes such as *c-myc* [21,22], *c-Kit* [23], *KRAS* [24], *VEGF* [25,26], *PDGF* [27], *BCL-2* [28], *c-Myb* [29], RET [30], AR [31], ADAM [32], hTERT [33], and MET [34], genes associated with telomere homeostasis [35], neurodegenerative diseases [36,37,38], mental retardation [39], involved in neoplasia [40], and untranslated regions [41]. Genes harboring putative G-quadruplex forming sequences (PQS) and related diseases are tabulated in Table 1. These G-rich regions and G-quadruplex structures are prone to chemically reactive molecules including ROS.

The increasing biological relevance of G-quadruplex entity in various biological processes and specifically their relationship with cancer diseases is well documented. The utmost importance is given to the investigation on the ROS caused lesions in guanine rich sequences. ROS can cause modification in guanines which could reduce the thermal stability of G-quadruplex motifs as well as affecting the binding of various G-quadruplex specific proteins [42]. This review is an attempt to mention the importance of the phenomenon of oxidative stress in biological system and its deleterious effect on the non-Watson–Crick structures like G-quadraplexes prone to be formed at G-rich genomic locations. This may add to the better understanding of the mechanism of oxidative stress and significance of repair pathways.

## 2. Role of ROS in Normal Cells

It is undeniable that an optimum level of ROS is required for the normal functioning of cell. It is required for cell signaling under both physiological as well as pathophysiological conditions. Overproduction of ROS is linked to various diseases but the indispensable role of ROS in redox homeostasis, the immune system, and facilitating various cellular functions cannot be ignored. Modulation of cell proliferation as well as apoptotic pathways for programmed cell death is also governed directly or indirectly via mediation of ROS [43]. A significant amount of ROS is required to regulate cell cycle and various signaling processes. Transcription nuclear factor-κB expression is also regulated by ROS. κB assists in the body’s inflammatory process via activation of monocyte chemotactic protein-1 (MCP-1) and interleukin-6 [44]. On invasion of foreign bodies in a cell, the immune system produces ROS which triggers phagocytosis of foreign bodies by macrophages, neutrophils, or dendritic cells [45]. Thus, the role of ROS in normal condition is unquestionable and their optimum level should be maintained in cellular environment.

## 3. ROS as Trigger of Oxidative Stress

The main wrongdoers of oxidative stress are the ROS, which can be produced exogenously as well as endogenously. Interestingly, the optimum levels of ROS are essential for the proper functioning of cells owing to their pivotal role in cell signaling and regulatory processes [46]. The exogenous source includes ionizing radiation, therapeutic agents, and environmental factors. ROS are continuously produced by radiolysis of water molecules as well as by product of secondary reactions which enhance ROS level resulting in prolonged toxicity in cell [47]. It has been proven that cancers in multiple target organs of human beings are induced by ionizing radiation and fatal carcinogens [48].

**Figure 1:**
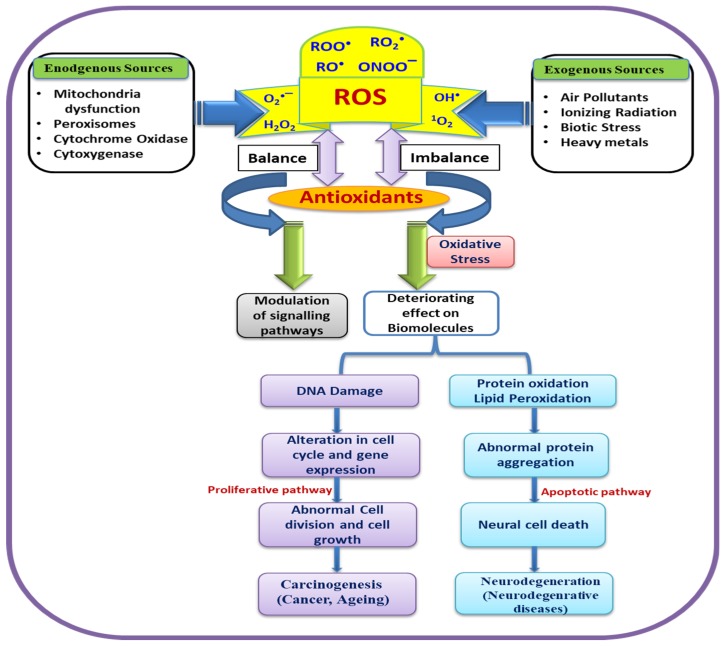
Schematic representation of biological role of reactive oxygen species (ROS) in oxidative stress and various diseases.

Environmental agents are also found to play a significant role in the induction of reactive oxygen species in cells [49]. A summarized schematic representation is depicted in Figure 1. These ROS significantly cause oxidative DNA damage, involved in carcinogenesis and aging along with alteration of gene expressions.

## 4. Oxidation Mechanism of Guanine Base via ROS

**Figure 2:**
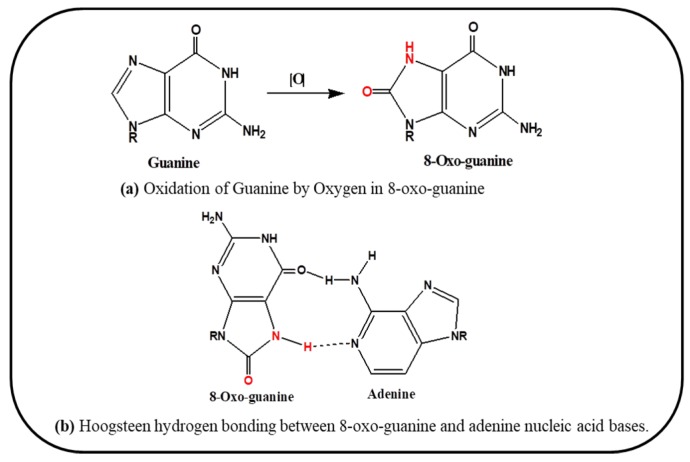
Schematic representation of mechanism of oxidation by oxygen (**a**) and base pairing via hydrogen bond between 8-oxo-guanine and Adenine (**b**).

Guanine, one of the four DNA nucleotide bases is more prone to oxidation, resulting in the formation of 8-oxo-7,8-dihydroguanine (8-oxo-dG). This is the most important oxidized DNA base being used as biomarker to detect involvement of oxidation in aging, cardiovascular diseases, neurodegenerative diseases and cancer [50,51,52,53,54]. This oxidized guanine is now available for pairing with Adenine via Hoogsteen hydrogen bonding in place of cytosine, leading to the mutation and genomic instability [51]. Figure 2 demonstrates the mechanism of oxidation of guanine by oxygen (a) the product 8-oxo-dG so formed is paired with Adenine (b).

Oxygen is a key component of cellular metabolism and plays a pivotal role in cell signaling and various biological processes. Apart from its beneficial role it is also suggested that through ROS generation it can interact with macromolecules (DNA, protein etc.) and cause severe damage to macromolecules and other cell components [55]. During biological processes a number of bond-makings and -breakings take place and via these an imbalance of electrons occurs, leading to the generation of free radicals. These free radicals come in contact with oxygen molecules, abundantly present in biological system, react with them, and generate ROS. The resultant ROS drastically affect the macromolecules by obstructing the proper synthesis and repair process of DNA. When the ROS level is elevated in the cellular environment, it inhibits the scavenging ability of antioxidants (some proteins and enzymes), resulting in an oxidative stress condition [56].

It is worth mentioning here that higher-energy singlet molecular oxygen (^1^O_2_) act site specifically i.e., it affects DNA bases (guanine) and amino acids (histidine, tyrosine, tryptophan) in various proteins selectively whereas O_2_^•−^ does not show any reactivity towards biomolecules, meaning it is inert towards biomolecules [57]. It is evident that organic molecules cannot react with molecular oxygen directly because of its existence in a singlet spin state whereas molecular O_2_ reside in the cell in the triplet spin state. Usually the reaction of triplet state with a singlet molecule is forbidden and takes place at very slow rate (<10^−5^ M^−1^·s^−1^). Various transition metals can act as bridges to overcome this barrier via reducing the molecular oxygen to free radical species. These free radical species then interact with organic molecules efficiently [58,59]. Among all the transition metals, iron plays a substantial role in catalyzing this reaction. Iron, being present at a higher concentration in biological systems, produces the oxygen radical. Other transition metals are also able to catalyze the reaction but at a slower rate and cause adverse effect to the biological system.

There are several mechanisms which include iron to produce reactive oxygen species. One such type of mechanism is depicted here involving various reactions in combination leading to the generation of a hydroxyl radical [60].

Reductant + Fe(III) → Oxidant + Fe(II)(1)

Fe(II) + O_2_ → Fe(III) + O_2_^•−^ (2)

HO_2_^•^ + O_2_^•−^ → O_2 +_ H_2_O_2_ (3)

Fe(II) + H_2_O_2_ → Fe(III) + OH^−^ + HO^•^ (4)

It is clearly seen here that hydroxyl radical (^•^OH) is generated by decomposition of H_2_O_2_ and the reaction is mediated through Fe (II) [Fenton reaction]. In spite of having very short half-life, ^•^OH is specifically very reactive and have tendency to damage DNA via the interaction of Fe(II)-ethylenediamminetetracetate (EDTA) as well as Fe(III)-nitrilotriacetate with H_2_O_2_ [61,62,63]. O_2_^•−^ usually exists as the unprotonated species in physiological conditions whereas in aqueous conditions it undergoes dismutation which results in the formation of H_2_O_2_ and O_2_. Owing to this, H_2_O_2_ is produced in good amounts as a byproduct along with the formation of O_2_^•−^. It has also been proven that enzymatic dismutation of O_2_^•−^ via superoxide dismutase is predominant over spontaneous dismutation at physiological pH. The rate of reaction of enzymatic dismutation is found to be four orders of magnitude faster than later. Interestingly, H_2_O_2_ which is produced as byproduct has no unpaired electron and cannot be categorized as a free radical. However, when it comes across redox-active transition metals it interacts with them, thus leads to the formation of ^•^OH [64]. It is well documented that ^•^OH can damage DNA in vivo involving Fenton reaction. ^•^OH can interact with DNA sugar-phosphate backbone and abstract a proton, leading to DNA cleavage at every nucleotide without showing any site specificity [65].

^•^OH radical reactions can take place via involving any of the three routes i.e., hydrogen abstraction, addition, and electron transfer. Many less reactive species are generated by these reactions. ^•^OH radicals efficiently react with DNA i.e. with all four bases, and deoxyribose, thus leads to deleterious effect on DNA. ^•^OH can abstract hydrogen from all the five carbons of deoxyribose and form carbon-centered radicals. These carbon radicals further either reacts with oxygen results in peroxyl radical or join together leads to the formation of nonradical products. In anaerobic conditions, β-cleavage takes place in C4′-centered radical results in DNA strand breakage and via this produce an intact base and altered sugar. In contrast, C1′-centered radicals undergo oxidation leading to the formation of sugar lactone formation along with release of an intact base. On the other hand, in the presence of oxygen, the addition of molecular oxygen takes place at a carbon-centered radical thus forms peroxyl radicals. As the result of this, carbon–carbon bond is cleaved and an alkali-labile site is produced. In another stance, β-cleavage and strand breakage takes place when a C5′-centered peroxyl radical converts to an oxyl radical, eventually leading to either the formation of an altered sugar along with an intact base or aldehyde formation at the C5′ end along with the strand breakage [66].

**Figure 3:**
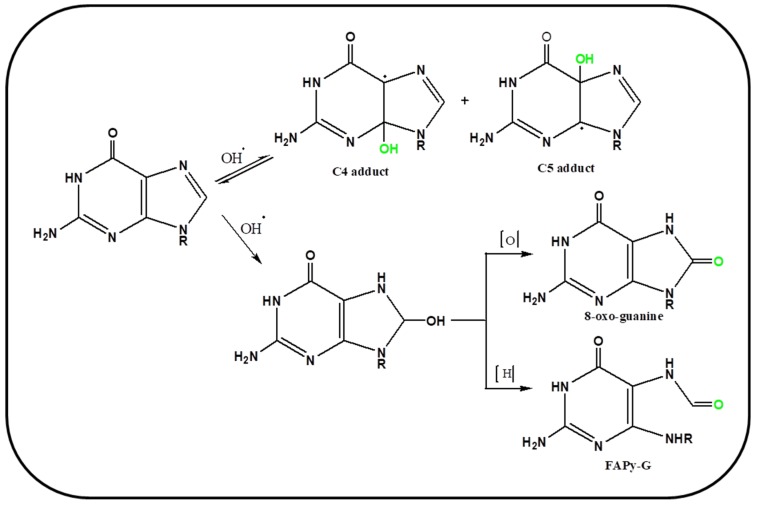
Schematic representation of mechanism of oxidation of guanine by hydroxyl radical.

OH can also produce various oxidation products by giving addition reaction with nucleic acid bases. It attacks guanine at three places namely C4, C5, and C8, where at two positions (C4 and C5) it shows reversible reaction. Two adducts formed as C4-adduct and C5-adduct are short lived, as soon as they receive an electron from the solution medium, it gets converted to guanine again and check/inhibit the permanent DNA damage. Whereas, adduct formed with C8 results in an intermediate having long half-life and thus it reacts with water and oxygen to produce 8-oxo-guanine and 2,6-diamino-5-formamido-4-hydroxypyrimidine (FAPy-G) [67]. Figure 3 shows the mechanism involving oxidation of guanine by hydroxyl radical. 8-oxo-G is well studied owing to its efficiency of pairing with Adenine and subsequently causes G→T transversion mutation in genome. The other purine i.e., adenine can also interact similarly with ^•^OH but the lesion caused by oxidation of adenine is not very prevalent in DNA damage and genomic stability.

Another important reagent causing DNA damage identified recently is known as peroxynitrite i.e., the reactive nitrogen species (RNS). It is associated with chronic inflammation and carcinogenesis. Phagocytes are present abundantly in biological system. Invasion and infection of any foreign body to the cell, leads to inflammation of tissues. As the result the phagocytes, in immune and inflammatory responses, produce NO and O_2_^−^ as cytotoxic agents. These entities react with each other and generate a highly reactive anionic species known as peroxynitrite (ONOO^−^) [68,69]. It plays a pivotal role in cellular signaling as well as it causes severe damage to nucleobases specifically guanine by converting it into nitration products.

**Figure 4:**
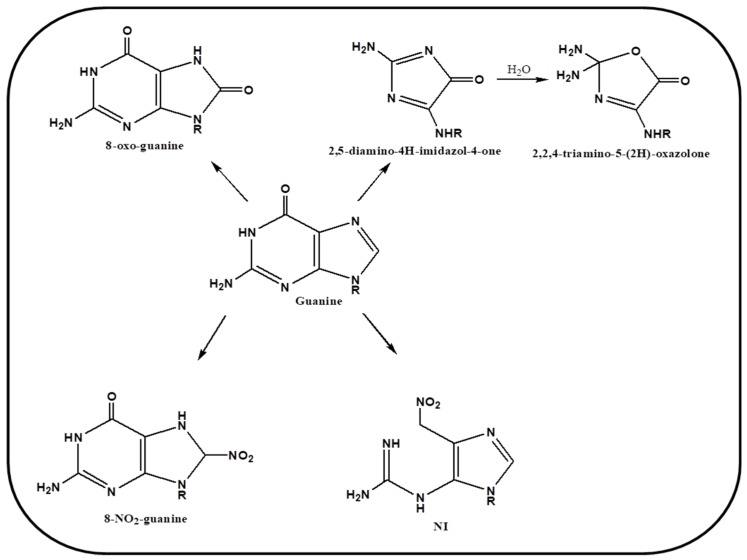
Schematic representation of mechanism of oxidation of guanine by peroxynitrite.

It was hypothesized earlier that 8-oxo-guanine can be generated by hydroxyl radical and nitro product by peroxynitrite, however later it was demonstrated that this RNS react with both guanine and 8-oxo-guanine product but reacts more efficiently with the later [70]. Various other secondary oxidative products are produced via interaction of peroxynitrite with 8-oxo-guanine such as 2,5-diamino-4H-imdazol-4-one (Iz) and 2,2,4-triamino-5-(2*H*)-oxazolone (Oz). A schematic representation of mechanism of oxidation by RNS is drawn in Figure 4. Tretyakova et al. have demonstrated that various secondary lesions are caused on nucleobase guanine by peroxynitrite, which is then recognized via Fpg repair enzyme [71]. Thus, various studies have confirmed a substantial role of ROS and RNS, in causing oxidative stress and DNA damage in biological systems, specifically causing guanine lesions in cellular environment. We further discuss the role of these species in guanine rich regions and their role in genomic stability.

## 5. G-Quartet Formation and Role of ROS

**Figure 5:**
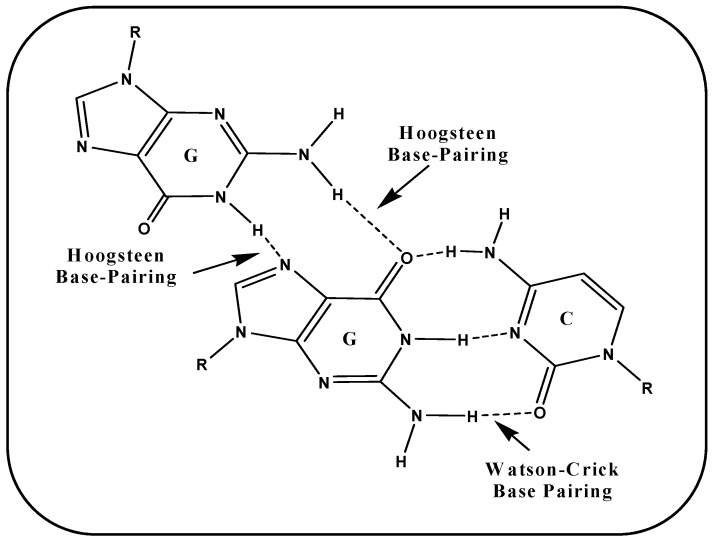
Base pairing scheme involved in G-G (Hoogsteen Base-pairing) and G-C (Watson–Crick Base-pairing).

A G-tetrad is formed by the association of four guanines paired via Hoogsteen hydrogen bonds. G-tetrads stack vertically one upon another to adopt a G-quadruplex structure linked by several loops. These Hoogsteen hydrogen base pairing is albeit different from typical Watson–Crick base pairing i.e., firstly it differs in the atom involved in hydrogen bonding, secondly in the number of hydrogen bonds via which nucleotides get paired. In case of G-G, Hoogsteen hydrogen bonds involve N7 and O6 of one face whereas N2 and N1 of another face. Figure 5 illustrated the base pairing scheme of Hoogsteen hydrogen bonding between G-G and Watson–Crick base pairing involved in G-C.

Absence of third hydrogen bond in Hoogsteen base pairing indicates the lesser stability of Hoogsteen hydrogen bonds than Watson–Crick base pairing. It is worth mention here that in G-tetrad total 8 hydrogen bonds are involved between four guanines which provides more stability to G-tetrad. G-tetrads arrange themselves one above the other to adopt secondary non-canonical G-quadruplex structures. On the basis of strand stoichiometry these can be tetramolecular (four strands), trimolecular (three strands), bimolecular (two strands), and unimolecular (one strand). Strand polarity also plays a pivotal role in determining the topology of G-quadruplexes i.e., parallel, antiparallel and mixed [19,72,73].

A central cavity is generated by the cyclic arrangement of guanine tetrads, where O6 atoms are arranged towards the center. Tetrads are stacked on one another so four O6 atoms are lined the cavity from one tetrad and four from the tetrad below or above to it. Oxygen is negatively charged so the cavity is neutralized and stabilized by monovalent cation. Cellular environment is rich in various cations. It is well documented that potassium having the best suitability towards the central cavity can effectively coordinate the oxygen atoms [74,75,76].

Literature is rich in the reports that guanine rich regions are abundantly present in telomeric ends as well as upstream promoter regions. These G-rich sequences have potential to adopt folded self-associated G-quadruplex structure. Enrichment of genome in these potential structures indicates towards their regulatory role in biological processes [77,78,79,80]. Recently in vivo evidence as well as their involvement in replication, transcription and recombination etc. is well established [19,81]. Various small molecules such as Quarfloxin, BRACO-19, Berberine, Telomstatin, and Sanguinarine [82,83] and proteins (Pif1 helicase, FMRP, RHAU etc.) specifically interact with these non-B-DNA structures in biological system to stabilize or destabilize these structures [84,85,86].

**Figure 6:**
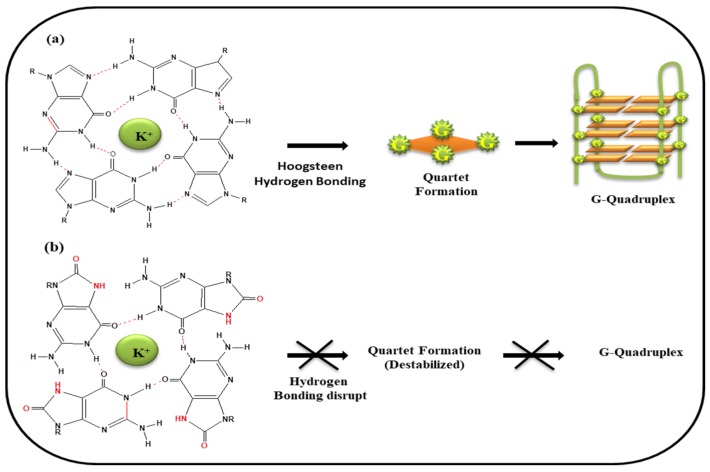
(**a**) Guanines paired via Hoogsteen hydrogen bonding leads to stabilized tetrad formation and thus G-quadruplex formation (**b**) on oxidation 8-oxo-Guanine formed, so the Hoogsteen Hydrogen bonding disrupted leads to destabilization of G-tetrad.

It has already been discussed in previous section that guanine having a low redox potential is most prone to oxidation. In view of this fact, guanine rich sequences, abundantly present at genomic locations may adopt G-quadruplex structure, and therefore also have potential to undergo oxidation and become vulnerable for oxidative stress. Figure 6a depicted the G-tetrad formation via 4 G^•^G Hoogsteen hydrogen bond and quartet stacked one above the other which in turn adopts folded G-quadruplex structure. G-quartet stabilized by harboring the K^+^ ion in the central core of the cavity. In presence of ROS, guanine converts into 8-oxo-Guanine and thus only two Hoogsteen hydrogen bonds are possible between two guanines leading to the obstruction of G-tetrad formation (Figure 6b).

Several groups have investigated the effect of ROS on the G-quadraplex formation with varied topology. In the following section we discuss the effect of ROS on the G-quadruplexes formed at telomeric and promoter locations, and their response towards oxidative damage.

### 5.1. (i) Telomeric G-Quadruplex and Role of ROS

Various research groups have investigated that guanine nucleobase is more vulnerable to oxidation. The telomeric 3′ overhang, at the end of chromosomes is a G-rich stretch of (TTAGGG)_n_ repeats prone to adopts G-quadruplex structure, is likely to a hub for oxidative damage [87,88]. It is interesting to observe the presence of 50% more of the oxidized guanine in quadruplexes in comparison to the duplex DNA [89]. Telomere shortening and premature senescence is caused due to the oxidative damage to telomeric G-rich sequences. Accordingly, telomeric region is more susceptible for oxidative stress in comparison to other single stranded and double stranded DNA. Vorlickova et al. has reported that guanine lesion via ROS can be possible in vivo which results in destabilization of G-quadruplexes structure nearly unfolded state at physiological conditions. Therefore, the unfolded strand is prone to be cleaved by cellular nucleases and if left unrepaired, results in telomere shortening [42]. Apart from end replication problem, telomere shortening is also induced by various ROS, causing oxidative damage due to single strand break. Various oxidizing agents induce single strand break (SSBs) in greater extent at telomeric DNA as compared to the DNA of other genomic locations [90,91,92,93,94,95,96,97,98].

Length of telomere is controlled and influenced by genetic as well as environmental factors. The telomerase, a ribonucleoprotein complex, regulates telomere-length maintenance and its integrity by adding telomeric repeats to the 3′-end. It is established that telomerase is actively involved in 85% of human cancer and tumor growth whereas it is inactive in somatic cells [99,100]. It has also been discussed previously that in presence of ROS guanine bases gets oxidized into 8-oxoguanine (8-oxo-G) and 5-hydroxy-methyluracil. The 8-oxo-Gunaine elevated level in telomere hinder the telomerase activity and obstruct the binding of telomeric protein to telomere DNA sequence, resulting in telomere shortening, function and its maintenance [101,102]. The loss of telomere end capping affected majority of cellular processes such as apoptosis, aging, carcinogenesis, and chromosomal instability [100]. When these lesions are not repaired properly, single and double strand breaks (SSBs and DSBs) are caused along with GC-TA mutations, further inducing the genomic instability [103]. Bielskute et al. has demonstrated that substituted guanine of telomeric oligonucleotide sequences with major product of ROS i.e., 8-oxo-7,8-dhydroguanine, reduce hydrogen bonding capability between G-tetrads and thus G-quadruplex structure. It was also shown that glycosidic conformation of G’s are also very important to combat oxidative lesions [104]. A recent report by Bozkus has highlighted the role of telomerase and serum levels of it as a marker of oxidative stress [105]. Aeby et al. recently studied and analyzed chromatin composition and demonstrated that telomeres are abundantly present in an antioxidant enzyme peroxiredoxin 1 (PRDX1) in the S phase of cell cycle. It was demonstrated that in absence of PRDX1 gene enable telomeric DNA prone to oxidative stress. This study revealed that this gene shield telomere from ROS as well as restrain it from telomere damage [106].

**Figure 7:**
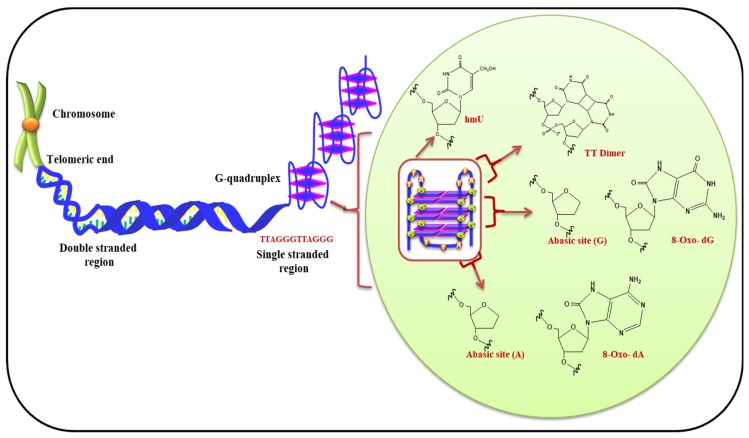
G-quadruplex formation at telomeric end and possible modification in DNA bases due to ROS.

Virgilio et al. have recently investigated effect of ROS on the bases present in loop of G-quadruplexes. It is demonstrated that the bases in loop have contrasting effect on stability of G-quadruplex. It was shown that the tetrad present in middle of G-quadruplex structure is less prone to oxidation in comparison to terminal tetrads. Figure 7 displayed the presence of telomeric G-quadruplex and modified bases in presence of ROS. This report also suggested the importance of nucleotides in loop and their substantial role in oxidative damage of G-quadruplex [107]. Therefore, the terminal G-tetrads shield the internal tetrads present in the quadruplexes. Fouquerel et al. and Sarkar et al. have recently given insight about the role of telomere length homeostasis. Their reports emphasized that in presence of increased levels of pre-existing 8-oxo-G (owing to defective OGG1 and ROS detoxification) telomerase-dependent telomere lengthening takes place [108,109].

Several laboratories worldwide are working for better understanding of the cause and consequences of oxidative stress on telomeric G-quadruplexes and its substantial role played in apoptosis, cellular senescence, carcinogenesis etc.

### 5.2. (ii) G-Quadruplexes in Promoter Region and Effect of ROS

Enrichment of Guanine rich sequences and G-quadruplex structures at promoter locations are well documented. Various oncogene promoters harbor G-rich stretches facilitating formation of non-canonical G-quadruplex structures [110]. The existence of G-quadruplex structures and their biophysical properties are well characterized at various functional regions (promoter, enhancer, 5′-UTR etc.). Involvement of these structures in transcription, translation, and gene regulation are well documented [111,112].

Oxidative DNA damage is well explained in literature specifically Guanine rich sequences and G-quadruplex structures. Fleming et al. have illustrated that a fifth G-track present near the G-quadruplex forming sequences is involved in the formation of these structure can act as a ‘spare-tire’ providing the platform for the oxidatively damaged Guanine stretches to bulge out in loop. These bulged-out Guanine stretches can behave as substrates for base excision repair enzymes. The existence and repair of these oxidatively-damaged guanine bases in promoter location might play a central role in epigenetic modification as well as regulation of gene expression [113].

In an another report Fleming et al. have again demonstrated that in the presence of ROS guanine is converted into 8-oxo-G and via this 8-oxoguanine glycosylase (OGG1) gets activated and induce base excision repair, resulting in the production of abasic sites (AP). The abasic site generated, unwinds the duplex to expose putative quadruplex sequences. Apurinic/apyrimidinic endonuclease 1 (APE1) binds to the so formed G-quadruplex and cleaves the AP which in turn facilitate the proper activation and smooth functioning of vascular endothelial growth factor (*VEGF*) or endonuclease III-like protein 1 (*NHL1*) genes [114]. Fedeles has recently suggested that these structural motifs control transcription process as counteract ion against the oxidative stress [17].

**Figure 8:**
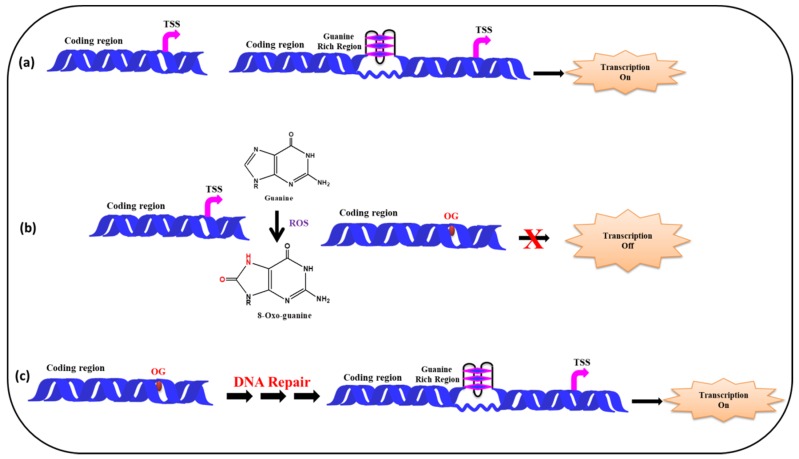
Effect of ROS on G-quadruplex formation and transcription. (**a**) In the absence of ROS, the G-quadruplex formation takes place activating the transcription (**b**) G-quadruplex formation is disrupted by ROS, as a result transcription is suppressed (**c**) DNA gets repaired and G-quadruplex formation leads to transcription.

Figure 8 depicts a model showing the presence of guanine rich sequences in coding region of a gene near transcription start site and via the formation of G-quadruplex transcription of the gene gets activated. In presence of ROS the guanine gets converted into 8-oxo-Guanine, disrupting the G-quadruplex formation which in turn inhibits transcription. Following the repair of the damaged Guanine, via cellular DNA repair mechanism (BER etc.) the G-quadruplex formation take place, further facilitating the smooth regulation of the transcription process.

Several reports are available revealing the association of oxidative DNA damage and cancer, as well as aging along with role of base excision repair in damaged DNA [53]. Byrd et al. have recently demonstrated the accumulation of G-quadruplexes as stress granule in cytoplasm against the oxidative stress. It is also shown that some proteins interact specifically to G-quadruplexes and modulate m-RNA translation which in turn facilitates the stress granule formation [115]. Fleming et al. reviewed it thoroughly, recently that various oxidation products (8-oxo-G, spiroiminodihydantoin (Sp), 5-guanidinohydantoin (Gh) etc.) formed in cell and cause lesions to secondary structure of DNA (double stranded DNA, G-quadruplex etc.). DNA glycosylases specifically identify these oxidation products in the cell, and BER is initiated via mammalian Nei-like1-3 (NEIL1-3) glycosylases [114]. Very recently, Shi et al. established that on ultraviolet light irradiation, poly-G-quadruplex-TMPyP4 complexes are prone to generate a high level of ROS. It leads to singlet oxygen production at cancer lesions and promotes apoptosis of malignant tissues. This study emphasized G-quadruplex-mediated photodynamic therapy targeting cancer cells [116]. A recent report has demonstrated that 8-oxo-G can play the role of an epigenetic marker for probing the employment of nuclear factors at promoter site substantial for *KRAS* transcription [117]. Thus with the prevalence of G-rich sequences at specific genomic locations, an interlacing between G-quadruplex and oxidative stress is well established.

## 6. Outlook

Oxidative stress research now encompasses cell biology, chemistry, biochemistry, physiology, and disease physiology to health and drug research. Major diseases, now even diabetes (type 2), are being considered as ‘redox diseases’ [118]. Oxidative stress is the precursor to oxidative damage. It can cause disruptions in normal mechanisms of cellular signaling and usually manifests into damaged DNA bases, protein oxidation, and lipid peroxidation products. Oxidative damage to DNA is of particular interest as it causes modification in DNA bases. However, owing to its low redox potential the guanine, among the four other nucleobases, is especially susceptible to oxidation. The range of G-rich sequences at genomic locations such as promoters, telomeric region, exons, enhancers, and immunoglobulin switch regions signifies their crucial role in various biological processes [119,120]. The formation of G-quadruplex structures harbor guanine in large numbers, making these structural forms subject to undergo oxidation at a greater extent. Oxidatively damaged G-quadruplexes may play a substantial role in down regulation of transcription process and gene regulation. The involvement of these non-Watson–Crick structures in diseases like neurodegenerative diseases, Alzheimer’s, and cancer is well established. Literature is rich in reports about deleterious effect of oxidation on DNA, causing damage; mutation etc., the same if left unrepaired further may lead to genomic instability.

The DNA damage on G-quadruplex (8-oxo-G) can possibly be utilized as biomarker or sensor to investigate the elevated level of ROS and thus oxidative stress in cellular environment. Specific proteins are identified which can exclusively recognize and repair these damaged guanines and facilitate G-quadruplex formation leading to smooth transcription and regulatory roles in biological system. Many of the antioxidant genes are known to be polymorphic, which can lead to altered enzyme activity. Since it is known that presence of G-rich segments on the promoter or coding region of the genes may facilitate the formation of quadruplex structures at physiological pH and salt conditions, the molecular insight will enhance the thrust of the concept of redox alterations due to oxidative stress. Due recognition of these structures at genomic locations by specific proteins (helicases) may modulate transcription, thus enabling them act as regulatory elements [121]. Though there have been various convincing reports regarding the pivotal role of oxidative stress in various biological processes involving G-quadruplexes, this review is a humble effort to give a glimpse about the role of these unusual structural forms of DNA in oxidative stress. Several research groups are active worldwide to congregate a more detailed molecular understanding and determine the exact role and mechanism of polymorphic G-quadruplex formation, as a response to stress into biology and medicine. Further studies would also deepen the translational impact of phenomenon of oxidative.

## Figures and Tables

**Table 1:** Human genes containing potential G-quadruplexe sequences, associated to various diseases.

S. No.	Gene	Disease	References
1	*c-Myc*	Gastrointestinal, ovarian and breast cancer tumors	[21,22]
2	*c-Kit*	Gastrointestinal stromal tumors (GIST)	[23]
3	*KRAS*	Pancreatic carcinoma	[24]
4	*VEGF*	Tumor angiogenesis	[25,26]
5	*PDGF*	Cancers and fibrotic disorders	[27]
6	*BCL-2*	B-cell and T-cell lymphomas and breast prostate cervical Colorectal and non-small cell lung carcinomas	[28]
7	C-Myb	Leukemias	[29]
8	RET	Thyroid cancers	[30]
9	AR	Castrate-resistant prostate cancer	[31]
10	ADAM	Breast cancer	[32]
11	hTERT	Limitless replication and cancer	[33]
12	MET	Cancers of kidney, liver, stomach, breast, and brain	[34]
13	BRCA2	Familial breast/ovarian cancer, telomere homeostasis	[35]
14	C9orf72 Gene	Amyotrophic lateral sclerosis (ALS) or frontotemporal dementia (FTD)	[36,37,38]
15	FMR1 Gene	Fragile X syndrome	[39]
16	ESR1	Cancer and neoplasia	[40]
